# Exploring the Anti-Diabetic Potential of Quercetagitrin through Dual Inhibition of PTPN6 and PTPN9

**DOI:** 10.3390/nu16050647

**Published:** 2024-02-25

**Authors:** Geetanjali B. Gone, Geonhui Go, Gibeom Nam, Woojoo Jeong, Hyemin Kim, Soah Lee, Sang J. Chung

**Affiliations:** 1 Department of Biopharmaceutical Convergence, Sungkyunkwan University, Suwon 16419, Republic of Korea; goneg@skku.edu (G.B.G.); hui1105@skku.edu (G.G.); skarlqja12@skku.edu (G.N.); gpals0506@skku.edu (H.K.); 2 School of Pharmacy, Sungkyunkwan University, Suwon 16419, Republic of Korea

**Keywords:** PTPN6, PTPN9, quercetagitrin, anti-diabetic, type 2 diabetes

## Abstract

Protein tyrosine phosphatases (PTPs) are pivotal contributors to the development of type 2 diabetes (T2DM). Hence, directing interventions towards PTPs emerges as a valuable therapeutic approach for managing type 2 diabetes. In particular, PTPN6 and PTPN9 are targets for anti-diabetic effects. Through high-throughput drug screening, quercetagitrin (QG) was recognized as a dual-target inhibitor of PTPN6 and PTPN9. We observed that QG suppressed the catalytic activity of PTPN6 (IC_50_ = 1 μM) and PTPN9 (IC_50_ = 1.7 μM) in vitro and enhanced glucose uptake by mature C2C12 myoblasts. Additionally, QG increased the phosphorylation of adenosine monophosphate-activated protein kinase (AMPK) and insulin-dependent phosphorylation of Akt in mature C2C12 myoblasts. It further promoted the phosphorylation of Akt in the presence of palmitic acid, suggesting the attenuation of insulin resistance. In summary, our results indicate QG’s role as a potent inhibitor targeting both PTPN6 and PTPN9, showcasing its potential as a promising treatment avenue for T2DM.

## 1. Introduction

Type 2 diabetes mellitus (T2DM), which is the most common type of diabetes, has become a major metabolic disease of clinical importance and has grown into a worldwide pandemic over the last few decades [1]. According to the International Diabetes Federation, the prevalence of T2DM is expected to increase to up to 783 million cases by 2045. T2DM is characterized by heightened levels of glucose and resistance to insulin in critical organs such as adipose tissue, liver, and skeletal muscles [2]. Comprising 51 amino acids, insulin serves a crucial function in maintaining glucose homeostasis, promoting cell growth, and regulating metabolism [3]. Insulin deficiency leads to impaired glucose uptake by cells, especially skeletal muscles. Hence, more glucose is observed in blood circulation [4]. Similarly, excessive consumption of fatty foods impairs insulin function by altering mitochondrial physiology [5]. Hence, patients with obesity and insulin resistance are expected to have excessive serum glucose and lipid build-up in their bodies [6]. Currently, synthetic compounds for the treatment of T2DM pose significant risks, including renal failure, cardiovascular complications, and hepatotoxicity [2,7]. Conversely, hypoglycemic agents derived from natural compounds are emerging as alternative therapeutics with lower toxicity and limited complicacies [8,9].

Protein tyrosine phosphatases (PTPs) constitute a specific category of enzymes critical to key cellular processes. The regular cell functions require the reversible and synchronized activity of protein tyrosine kinases (PTKs) and PTPs. The abnormal functioning of PTPs interferes with PTK activity by eliminating phosphate groups from the target proteins via hydrolysis [10,11]. Several human diseases, including T2DM, are associated with dysregulated PTP activities [12,13]. Therefore, PTPs are gaining recognition as potential targets for treating T2DM [14]. For example, PTPN1, PTPN2, PTPN11, PTPN6, DUSP9, and PTPRS have been shown to disrupt the insulin signaling pathways that are essential for regulating glucose homeostasis [2,13,14,15,16]. Two pathways have been implicated in diabetes development. The first pathway is Akt signaling, in which high blood sugar levels cause insulin to bind to insulin receptors and activate its substrate (IRS)-1. Consequently, this triggers downstream signaling through phosphoinositide (PI) 3-kinase (PI3K) and Akt pathway [3,6]. The second pathway is the adenosine monophosphate-activated protein kinase (AMPK) signaling pathway, which corresponds to insulin-independent. AMPK is a paired peptide kinase and its role in glucose homeostasis has been well studied [2,14,17,18]. These pathways facilitate glucose uptake by activating the migration of glucose transporter type 4 (GLUT-4) to the plasma membrane [19,20,21].

Previously, we demonstrated that knocking down PTP non-receptor type 6 (PTPN6) and non-receptor type 9 (PTPN9) [7,13] elevated glucose absorption in mature C2C12 myoblasts and 3T3-L1 white adipocytes through the stimulation of Akt or AMPK. This finding suggests that inhibiting these specific PTPs holds promise as a strategic approach for facilitating glucose homeostasis. This motivated us to identify natural therapeutic agents that can serve as a dual inhibitor of PTPN6 and PTPN9.

We previously conducted high-throughput screening of natural compound libraries (Figure S1). Among 1033 compounds, quercetagitrin (quercetagetin-7-O-glucoside, QG) repressed the catalytic action of PTPN6 and PTPN9 in vitro, suggesting its role as a dual inhibitor of PTPN6 and PTPN9. QG is a flavonoid derivative, obtained through extraction from marigold plants (*Tagetes erecta*). Flavonoids are phenolic secondary metabolites that have demonstrated widespread pharmacological properties, most importantly anti-diabetic and anti-inflammatory properties [22]. However, the specific anti-diabetic effect of QG and its downstream signaling remains uncertain.

In this investigation, we evaluated two effects of QG; its ability to hinder the catalytic activity of PTPN6 and PTPN9, and its capacity to enhance glucose uptake in C2C12 myoblasts. Furthermore, we examined the impact of QG on stimulating Akt and AMPK signaling in C2C12 myoblasts.

## 2. Materials and Methods

### 2.1. PTPN6 and PTPN9 Overexpression and Purification

The purification and expression techniques for PTPN6 and PTPN9 have previously been outlined in literature [7,13,15]. Escherichia coli Rosetta (DE3) cells were utilized to express recombinant plasmids encoding PTPN6 and PTPN9, supplied by Merck KGaA, Germany. The expression of PTPN6 and PTPN9 was achieved by exposing the cells to 0.1 mM isopropyl β-D-1-thiogalactopyranoside (IPTG) for a period of 24 h at a temperature of 18 °C. Following expression, cells were collected via centrifugation at 3570× *g* for 15 min at 4 °C, then resuspended in lysis buffer before being disrupted through ultrasonication. The lysate was then centrifuged at 29,820× *g* for 40 min at 4 °C to separate the supernatant, which was subsequently incubated using cobalt affinity resin (TALON^®^; Takara Korea, Seoul, Republic of Korea) and agitated on a rocker at 4 °C. The resin underwent a wash phase with 10 mM imidazole in a lysis buffer, after which PTPN6 and PTPN9 were isolated with a lysis buffer comprising 100 mM imidazole. Finally, the purified PTPN6 and PTPN9 proteins were concentrated and stored at −70 °C for further use.

### 2.2. Assessment of Enzymatic Activities, Half-Maximal Inhibitory Concentration (IC_50_) Values, and Hill Coefficients

The catalytic actions of PTPN6 and PTPN9 were assessed through the common PTP substrate, DiFMUP (6,8-difluoro-4-methylumbelliferyl phosphate). Techniques for calculating the catalytic activity were performed as per previous studies [2,7]. The kinetic constants were determined by adding PTPN6 (6 nM) and PTPN9 (0.05 nM) into a reaction mixture composed of 20 mM Bis-Tris (pH 7.0 for PTPN6) or 20 mM Bis-Tris (pH 6.0 for PTPN9), 150 mM NaCl, 0.01% Triton X-100, and 2.5 mM dithiothreitol (DTT), with varying DiFMUP concentrations (800, 400, 200, 100, 50, 25, 12.5, or 6.25 μM), reaching a total volume of 100 μL in a black 96-well plate. The fluorescence generated by the enzyme-substrate interaction was continuously recorded (excitation/emission = 355/460 nm) for 10 min using a Victor™ X4 microplate reader (Perkin Elmer, Norwalk, CT, USA). The kinetic constants, including the Michaelis constant (*K*_M_), maximum reaction velocity (*V*_max_), and turnover number (*k*_cat_), were derived from the analysis of Lineweaver–Burk and Michaelis–Menten plots (Figures S2 and S3). To ascertain the half inhibitory concentration of QG for PTPN6 and PTPN9, various concentrations of QG (8, 4, 2, 1, 0.5, 0.25, and 0.125 μM for PTPN6 and 10, 5, 2.5, 1.25, 0.625**,** and 0.3125 μM for PTPN9) were incubated with 2 × *K*_M_ (371.4 μM for PTPN6 and 314.8 μM for PTPN9. IC_50_ values were computed using Prism 10 (GraphPad Software Inc., San Diego, CA, USA) based on the sigmoidal dose-response curve. Furthermore, the Hill coefficients (n_H_) for both enzymes, indicating the degree of cooperativity between QG and the PTPs, were ascertained from the Hill plot (Slopes), employing the Hill equation [23].

### 2.3. Cell Culture and Cell Differentiation

The methodology for culturing and differentiating C2C12 cells has been documented in earlier studies [16]. In brief, C2C12 myoblasts were acquired from the American Type Culture Collection (CRL-1772, ATCC; Manassas, VA, USA). These cells were grown in a high-glucose Dulbecco’s Modified Eagle Medium (DMEM; LM001-07, Welgene Inc., Gyeongsan-si, Republic of Korea) enriched with 20% fetal bovine serum (FBS; S 001-01, Welgene, Gyeongsan-si, Republic of Korea) and an antibiotic-antimycotic mix (LS 203-01, Welgene, Gyeongsan-si, Republic of Korea). The culture environment was kept at 37 °C with 5%. For differentiation, once C2C12 muscle cells reached full confluency (100%), they were transitioned to a high-glucose DMEM containing 2% horse serum (16050-130, Thermo Fisher Scientific Korea Ltd., Seoul, Republic of Korea) along with the antibiotic-antimycotic solution and allowed to differentiate over a period of four days.

### 2.4. Cell Cytotoxicity Assay

The cell viability assay was conducted following the full differentiation of C2C12 myoblasts as previously described [15,16]. Upon achieving complete differentiation, cells were subjected to a 16 h starvation period in low-glucose DMEM (11885084, Gibco BRL, Middlesex, UK). Following this period of starvation, the cells underwent treatment with varying concentrations of QG (10, 20, 30, and 40 μM) in a no-glucose medium (11966025, Gibco BRL, Middlesex, UK) for a duration of 6 h. To evaluate the impact of these treatments on cells, a cell viability assay using EZ-cytox kit (EZ-500, EZ-Cytox, Daeil Lab Service, Seoul, Republic of Korea) was conducted. The viability of the cells post-treatment was determined by measuring the absorbance at 450 nm using the Victor™ X4 plate reader.

### 2.5. Glucose Uptake Assay

Glucose absorption was assessed using the protocol previously outlined in the literature [7,15]. Briefly, well-differentiated C2C12 myoblasts underwent a 16 h starvation phase in low-glucose DMEM (11885084, Gibco BRL, Middlesex, UK) to deplete essential nutrients. Subsequently, these cells were exposed to various QG concentrations for 6 h, followed by a 30 m treatment with 50 nM insulin in a no-glucose medium (11966025, Gibco BRL, Middlesex, UK) to stimulate glucose uptake. After the treatment, the cells were washed twice with 1 × PBS (LB 001-01, Welgene, Gyeongsan-si, Republic of Korea) and then incubated with a 100 μM solution of the fluorescent glucose analog, 2-[N-(7-nitrobenz-2-oxa-1,3-diazol-4-yl) amino]-2-deoxyglucose (2-NBDG; N13195, Thermo Fisher Scientific, Waltham, MA, USA), for 90 min. The level of glucose uptake by the cells was measured by detecting the fluorescence at an excitation/emission wavelength of 465/540 nm using a Victor^TM^ X4 plate reader.

### 2.6. Palmitic Acid-Induced Insulin Resistance in C2C12 Myoblasts

Conjugates of palmitic acid and bovine serum albumin (BSA) were prepared following a method as outlined earlier [24], with minor adjustments. The methodology for evaluating palmitic acid-induced insulin resistance has been previously outlined [16]. In this approach, sodium palmitate (P9767, Sigma-Aldrich, St. Louis, MS, USA) was solubilized in 150 mM NaCl at a temperature of 70 °C and combined with fatty acid-free BSA (A7030, Sigma-Aldrich, St. Louis, MS, USA), which was previously solubilized in 150 mM NaCl at 30 °C with a 10% (*w*/*v*) concentration, achieving a pH of 7.4. This solution was then stirred and passed through a 0.22 µm filter, resulting in palmitic acid–BSA conjugates with a 7.5 mM concentration at a 1:5 molar ratio. To induce insulin resistance, mature C2C12 myoblasts underwent a 3 h starvation period in serum-free DMEM (LM001-07, Welgene, Gyeongsan-si, Republic of Korea) followed by 16 h incubation with 200 µM of the palmitic acid–BSA conjugate in the same medium. The QG treatment group was treated with 40 µM QG concentration followed by 50 nM insulin for 30 min as per the previously mentioned protocol. 

### 2.7. Western Blotting

Western blotting analysis was conducted following a well-established protocol [7]. In summary, proteins were separated on a 10% sodium dodecyl sulfate-polyacrylamide gel (SDS-PAGE) and subsequently transmitted to a PVDF (polyvinylidene fluoride) membrane (Merck KGaA, Darmstadt, Germany) through a wet blotting technique. To prevent non-specific binding, 5% blocking solution made with skimmed milk was applied to the membrane. The membranes were subsequently left to incubate overnight at 4 °C with primary antibodies, including anti-total-AMPK, anti-phosphorylated AMPK-alpha (T172), anti-total- Akt, anti-phosphorylated Akt (S473) (Cell signaling Technology, Danvers, MA, USA), and anti-β-actin (AbFrontier, Seoul, Republic of Korea). Following primary antibody incubation, the membranes were treated with secondary antibodies specific to rabbit IgG coupled with horseradish peroxidase (AbFrontier, Seoul, Republic of Korea). The detection of the bound antibodies was facilitated by the EzWestLumi Plus kit (ATTO Corporation, Tokyo, Japan) and visualized using the LuminoGraph II system (ATTO Corporation, Tokyo, Japan) for chemiluminescence.

### 2.8. Docking Study of QG on PTPN6 and PTPN9

Ligand docking was performed using the Schrödinger Maestro 2020-4 software, following established methodologies on a Windows 10 platform [25]. The molecular structure of QG was retrieved as an SDF file from the PubChem database (https://pubchem.ncbi.nlm.nih.gov/ (accessed on 10 January 2024)) and prepared with the LigPrep tool within Schrödinger. Various ionization states of the ligands, including their neutral forms, were generated at a physiological pH to be used in the docking studies. The crystallographic structure of PTPN6 (SHP1, PDB ID: 3PS5) and PTPN9 (PTP-MEG2, PDB ID: 4GE6) were sourced from the Protein Data Bank (https://www.rcsb.org/ (accessed on 10 January 2024)) [26,27]. These protein structures were then prepared using Schrödinger’s protein preparation wizard, which included tasks such as adding missing side chains and removing water molecules and other non-essential entities. The docking grids were centered on the active sites as indicated by the position of co-crystallized inhibitors, with grid dimensions set to 25 Å. The Glide module in extra precision (XP) mode was employed for docking, adhering to the default parameters without applying any specific constraints. For each ligand, twenty docking poses were generated to allow for comprehensive analysis.

### 2.9. Statistical Significance Analysis

Statistical analysis to determine significance (*p* < 0.05) involved conducting a one-way ANOVA for comparisons among multiple groups, followed by the Tukey–Kramer method, and the implementation of a two-tailed unpaired t-test for comparing two groups, all performed using Prism 10 (GraphPad Software Inc., San Diego, CA, USA).

## 3. Results

### 3.1. Docking Model Predicts the Binding of QG to the Catalytic Sites of PTPN6 and PTPN9

**Figure 1:**
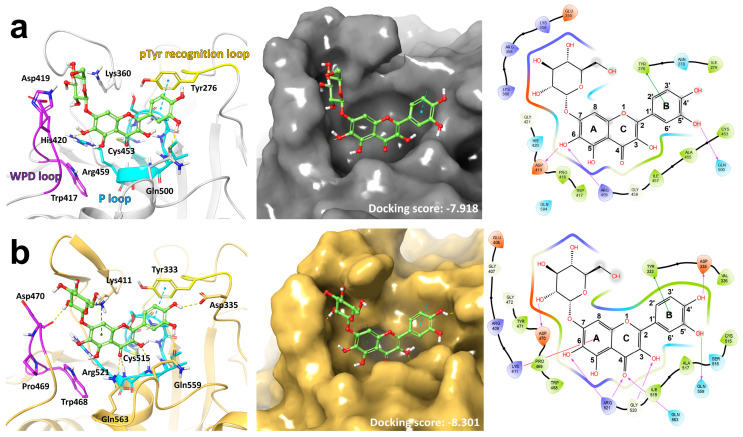
Docking model of quercetagitrin (QG). Ligands are depicted using ball−and−stick models, while critical motifs and residues of PTPN6 are emphasized with color. Hydrogen bonds are illustrated with yellow dashed lines. π–π stacking and π–cation interactions are represented as blue and green dashed lines, respectively (**left**). (**a**) Model of QG (pale green) on PTPN6 (white, PDB ID: 3PS5). The PTPN6 surface is shown in gray (**center**). 2D diagram of docking model (**right**). (**b**) Model of QG on PTPN9 (gold, PDB ID: 4GE6, **left**). The PTPN9 surface is shown in gold (**center**) and 2D diagram of docking model (**right**).

A computational docking study of QG with PTPN6 and PTPN9 was conducted. The catalytic sites and their signature motifs, including PTPN6 and PTPN9, are conserved in most PTPs. The catalytic sites of the PTPs exhibit high conservation and feature distinct motifs. The catalytic cysteine and HCX5R motifs within the P-loop of the catalytic site are crucial for substrate binding and catalytic dephosphorylation. Another conserved motif is the WPD loop [14,28]. Upon binding of the ligand to the catalytic site, the dynamic WPD loop envelops both the P-loop and ligand, triggering the catalytic reaction by introducing an aspartate residue [29,30]. The docking models of QG show that the poses of PTPN6 and PTPN9 share several similar features. The hydroxyl and carbonyl groups at the 3-, 4-position of the flavone moiety, are located in the P-loop (Figure 1a,b). The arginine residue of the P-loop also forms an H-bond with QG. The 3′,4′-dihydroxyphenyl group forms π–π stacking with the tyrosine residue of the pTyr recognition loop. The sugar moiety of QG is located in the WPD loop and forms H-bonds. Lys411 of PTPN9 formed a π–cation interaction with the A-ring of the flavone moiety. Our docking study suggests that the dual activity of QG on PTPN6 and PTPN9 results from the structural similarity between their catalytic sites.

### 3.2. QG Inhibits Catalytic Activity of PTPN6 and PTPN9 In Vitro

**Figure 2:**
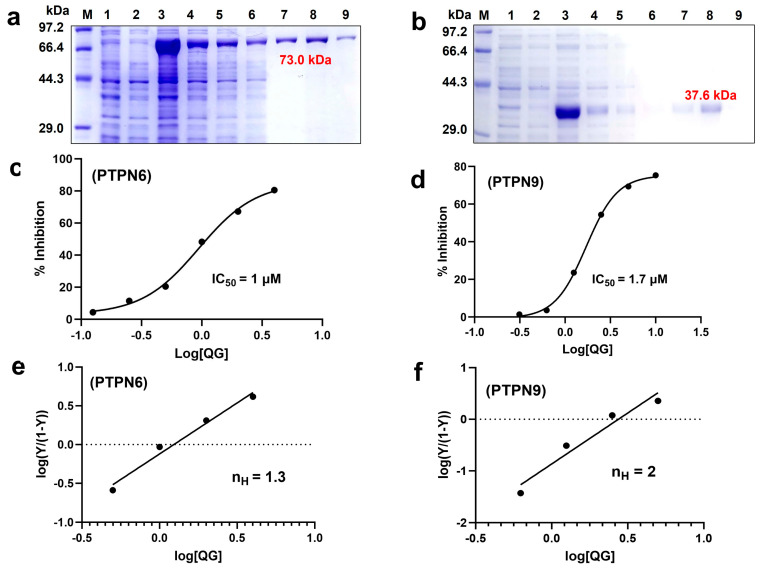
Quercetagitrin (QG) effectively hinders the catalytic action of both PTPN6 and PTPN9. (**a**,**b**) SDS−PAGE analysis of PTPN6 (**a**) and PTPN9 (**b**) with molecular weights of 73.0 kDa and 37.6 kDa respectively. M, protein molecular weight marker; 1: total cell lysate before induction; 2: supernatant before induction, 3: total cell lysate after induction, 4: supernatant after induction; 5: the sonicated sample flowed through the resin; 6: Sample collected post-column washing with lysis buffer; 7: Sample collected post−column washing with lysis buffer that includes 10 mM imidazole; 8 and 9: protein extracted with lysis buffer containing 100 mM imidazole. (**c**,**d**) IC_50_ values for QG, determined by % inhibition against log [QG] (μM) using a sigmoidal graph (**e**,**f**) n_H_ values in Hill plots are calculated from the slopes obtained through the Hill equation.

PTPN6 and PTPN9 are recognized as potential T2DM targets. Inhibition of these PTPs increases phosphorylated AMPK and insulin receptor (IR) signaling [13,15]. In this study, PTPN6 and PTPN9 were confirmed as viable targets for the treatment of T2DM through the identification of the inhibitory effects of QG on their enzymatic activity. First, *E. coli* was utilized for the overexpression of PTPN6 and PTPN9, followed by purification utilizing affinity resin (Figure 2a,b). Required kinetic constants of PTPN6 and PTPN9 were determined (Table 1) and used to determine the IC_50_ of QG against PTPN6 (IC_50_ = 1 μM) and PTPN9 (IC_50_ = 1.7 μM) (Figure 2c,d). Additionally, the extent of collaboration between QG and PTPs was evaluated by employing the Hill coefficient (n_H_), where an n_H_ value greater than 1 indicates positive cooperativity, n_H_ equal to 1 suggests no cooperativity and an n_H_ less than 1 denotes negative cooperativity [23]. The n_H_ values for PTPN6 and PTPN9 were 1.3 and 2, respectively (Figure 2e,f), indicating positive cooperation. The findings suggest that QG hinders the enzymatic function of PTPN6 and PTPN9. 

### 3.3. QG Facilitates Glucose Uptake in C2C12 Myoblasts

**Figure 3:**
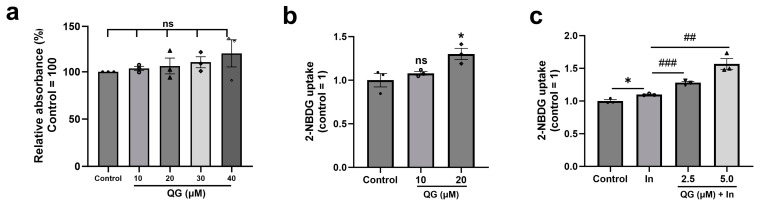
Quercetagitrin (QG) does not exhibit cell cytotoxicity, and it promotes increased glucose uptake. (**a**) Cell viability was assessed after complete differentiation of C2C12 myoblasts. The cells were subjected to starvation with low glucose media for 16 h. Followed by treatment with 10, 20, 30, and 40 μM QG and control, 0.1% MDSO in no glucose DMEM for 6 h. Well-differentiated C2C12 myoblasts (**b**) were exposed to 10 and 20 μM QG, alongside the control group treated with 0.1% DMSO, for 6 h. (**c**) were incubated with control (0.1% DMSO), 50 nM insulin (positive control, indicated as In), or co-treated with 2.5, 5 μM QG (6 h), and 50 nM insulin (30 min). Further, cells were treated with a 2-NBDG for 90 min to monitor the glucose uptake. Results are represented as the mean ± standard deviation. ns is considered as non-significant compared to the control group, * *p* < 0.05 is considered significantly different compared to the control group, ## *p* < 0.01, and ### *p* < 0.001 is considered significantly different compared to insulin only group.

In T2DM, hyperglycemia leads to the dysregulation of insulin-mediated glucose homeostasis in insulin-susceptible tissues, for example, myocytes and adipose tissue [31,32]. Previous studies have shown that PTPN6 and PTPN9 inhibitors facilitate glucose uptake and exhibit anti-diabetic activity [11,12,13]. Thus, the study aimed to evaluate the effect of QG on glucose uptake, either independently or in conjunction with insulin. First, the optimal QG concentration for treating C2C12 myoblasts was determined using a cell cytotoxicity assay. For the cell cytotoxicity assay, the C2C12 muscle cells were differentiated and treated as per the mentioned protocol. QG did not show any cell cytotoxicity in differentiated C2C12 myoblasts (Figure 3a). To clarify the anti-diabetic influences of QG, 2-NBDG, a fluorescent glucose probe, was used to measure glucose uptake in mature C2C12 myoblasts. Well-differentiated C2C12 myoblasts underwent treatment using 10 and 20 μM QG, or control, 0.1%DMSO (Dimethyl Sulfoxide). QG significantly increased glucose uptake in C2C12 myoblasts compared to the control (Figure 3b).

We then examined whether QG could further increase glucose absorption in the presence of insulin. Differentiated C2C12 myoblasts treated with insulin showed a significant increase in glucose uptake (*p* = 0.0130; Figure 3c). In the presence of insulin, the addition of QG resulted in a dose dependent increase in glucose uptake. Specifically, 5.0 µM QG treatment together with insulin led to 1.4-fold (*p* = 0.0048) increase in 2-NBDG uptake compared to the insulin only group (Figure 3c), indicating the additive anti-diabetic effect of QG when co-treated with insulin. To summarize, quantitative analysis revealed that QG effectively augments glucose absorption, exhibiting efficacy both in isolation and synergistically with insulin.

### 3.4. QG Increases AMPK Phosphorylation in C2C12 Myoblasts

**Figure 4:**
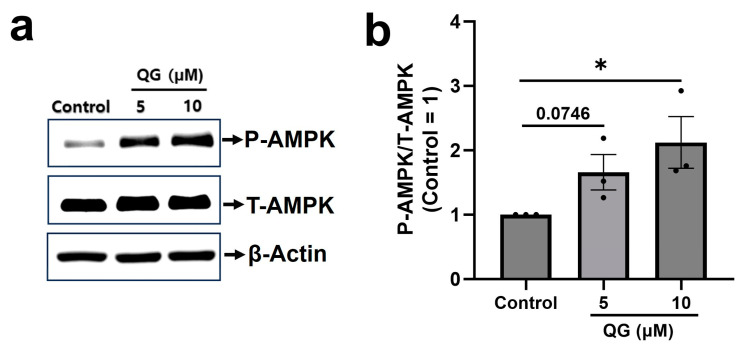
Quercetagitrin (QG) increases AMPK phosphorylation in C2C12 myoblasts. (**a**) Differentiated C2C12 myoblasts were treated with 5 and 10 μM QG (6 h) followed by western blotting using antibodies targeting phosphorylated AMPK (P−AMPK), total−AMPK (T−AMPK), and β−actin. (**b**) Quantitative analysis of P−AMPK/T−AMPK levels utilizing the image analysis software (ATTO Corporation, Tokyo, Japan). Results are represented as the mean ± standard deviation (*n* = 3). * *p* < 0.05 was considered considerably different in comparison to the control group.

Increased AMPK phosphorylation is an important molecular signature of the anti-diabetic activity of therapeutic phytochemicals [33]. Therefore, the effects of QG treatment on AMPK phosphorylation were analyzed. In this study, differentiated C2C12 myoblasts were evaluated. Our investigation revealed that treatment with 5 and 10 μM QG notably enhanced AMPK phosphorylation (*p* = 0.076 and *p* < 0.05 respectively; Figure 4a,b). These findings suggest that QG successfully increased AMPK phosphorylation.

### 3.5. QG Increases Insulin-Dependent Phosphorylation of Akt in C2C12 Myoblasts

Canonical insulin signaling involves the phosphorylation of Akt, which eventually manages to increase glucose uptake. Therefore, we investigated whether QG treatment synergizes with insulin in regulating Akt phosphorylation [14,34]. In this study, differentiated C2C12 myoblasts were treated with QG, with or without insulin.

**Figure 5:**
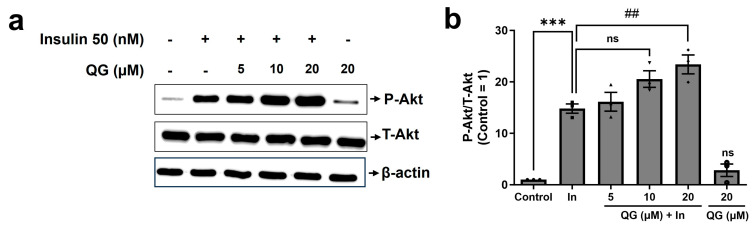
Quercetagitrin (QG) demonstrates an insulin-sensitizing effect by stimulating the phosphorylation of Akt. (**a**) Differentiated C2C12 myoblasts were treated with the control (0.1% DMSO, 50 nM insulin (as a positive control, represented as In), co-treatment with 5, 10, or 20 μM QG (6 h) and 50 nM insulin (30 min), or 20 μM QG alone (6 h), followed by Western blotting using antibodies targeting phosphorylated Akt (P−Akt), total Akt (T−Akt), and β−actin. (**b**) Quantitative analysis of P−Akt/T−Akt levels was performed using the image analysis software (ATTO Corporation, Tokyo, Japan). Results are represented as the mean ± standard deviation. *** *p* < 0.001 were considered significantly different compared to the control group, ## *p* < 0.01 were considered significantly different compared to the positive control group, and ns is considered as non-significant compared to the positive control group.

Insulin treatment significantly increased the phosphorylation of Akt in myoblasts by approximately 15-fold (*p* = 0.0002; Figure 5a,b). By contrast, the QG treatment only group led to no prominent changes in phosphorylated Akt expression (*p* > 0.05). In myoblasts, QG markedly influenced insulin-dependent phosphorylation of Akt in a concentration-dependent way. Specifically, at 20 μM dosage QG increased Akt phosphorylation by 4.8-fold (*p* = 0.0091) when compared to the insulin only treatment. These results suggest a synergistic effect between QG and insulin.

### 3.6. QG Mitigates Palmitic Acid-Induced Insulin Resistance in C2C12 Myoblasts through the Insulin-Dependent Akt Pathway

**Figure 6:**
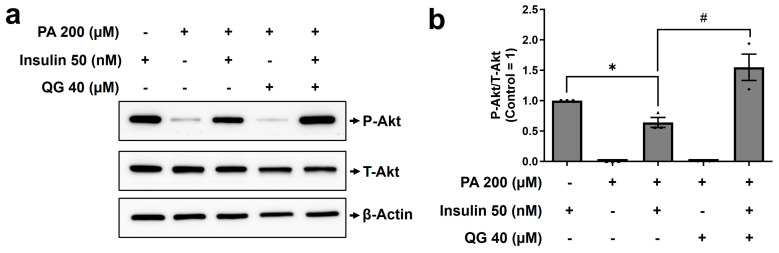
Quercetagitrin (QG) stimulates insulin-dependent Akt phosphorylation in cells with Palmitic acid−induced insulin resistance. (**a**) Mature C2C12 myoblasts were treated with 200 μM palmitic acid (PA, 16 h) followed by 40 μM QG treatment (6 h) and 50 nM insulin (15 min). The western blotting analysis was performed using antibodies targeting phosphorylated Akt (P−Akt), total Akt (T−Akt), and β−actin (**b**) Quantitative analysis of P−Akt/T−Akt levels was performed using the image analysis software (ATTO Corporation, Tokyo, Japan). Results are represented as the mean ± standard deviation (*n* = 3). * *p* < 0.05 was considered substantially different compared to the control group and # *p* < 0.05 was considered substantially different compared to the insulin-resistant group.

The intake of free fatty acids, notably palmitic acid, has been closely linked to the development of insulin resistance and a reduction in glucose uptake in muscle cells [16,35]. Whether QG confers a preventive response against palmitic acid-induced insulin resistance in differentiated C2C12 myoblasts was investigated. First, the differentiated C2C12 myoblasts were pretreated with 200 μM palmitic acid to induce insulin resistance. We found a 40% reduction in Akt phosphorylation compared to that in the insulin only group. Notably, co-treatment with insulin and QG led to an approximately 3-fold (*p* = 0.0172) increase in Akt phosphorylation in C2C12 myoblasts compared to that in the insulin-resistant group (insulin + palmitic acid group); Figure 6a,b). In summary, QG alleviated insulin resistance induced by palmitic acid in C2C12 myoblasts by enhancing the phosphorylation of Akt. 

## 4. Discussion

T2DM is a lifestyle disorder and can be initially managed with lifestyle changes and exercise; however, in chronic cases, medicinal intervention may be necessary. Although synthetic medications are used to target T2DM, their prolonged use is associated with various side effects and complexities [36,37]. By contrast, plant-based bioactive compounds exhibit potent pharmacological actions and are free of undesirable side effects [38,39,40].

QG is a natural flavone-based compound best known for its neuroprotective properties. Although its parent compound, quercetagetin, exhibits strong anti-diabetic properties [41,42,43], no studies have been performed to confirm QG’s anti-diabetic activity and related signaling pathways. PTPs have been identified as potential drug targets in T2DM [14]. Specifically, the knockdown of PTPN6 and PTPN9 effectively increased glucose uptake via AMPK phosphorylation [2,7,13,15], motivating the discovery of potential natural drug compounds to be used against them.

Given the structural similarity of QG to flavonoids and its inhibitory effect on PTPN6 and PTPN9, we hypothesized that QG would exhibit anti-diabetic activity. To identify a natural drug compound that inhibits the enzymatic activities of both PTPN6 and PTPN9, high-throughput screening of an in-house library of 1033 natural drug compounds was performed. QG was revealed as a promising drug for targeting PTPN6 and PTPN9 (Figure S1). Docking simulation predicted the binding of QG to the catalytic motifs of both PTPN6 and PTPN9 (Figure 1). Kinetic studies, together with the docking model simulation, suggested that the binding of QG to PTPN6 and PTPN9 leads to the inhibition of their catalytic activity (Figure 2). Furthermore, QG treatment led to significant glucose uptake in C2C12 myoblasts, which further increased upon co-treatment with insulin, indicating a synergy between insulin and QG (Figure 3). Collectively, these results imply that QG can upregulate glucose uptake through the catalytic inhibition of PTPN6 and PTPN9.

Dysregulation of PTPs downregulates AMPK and Akt phosphorylation [12], affecting glucose homeostasis maintained by both insulin-independent, i.e., AMPK, and insulin-dependent, i.e., Akt, pathways [14]. In our experimental confirmation using C2C12 myoblasts, a remarkable upregulation in phosphorylated AMPK expression was noticed after the treatment with QG for 6 h (Figure 4). Furthermore, our results indicated that QG upregulated Akt phosphorylation in the presence of insulin, highlighting its potential role in activating insulin-dependent pathways (Figure 5). A previous study exhibited that the ethanolic extract of *Ficus tikoua* improved glucose uptake through the AMPK and PI3K/Akt pathways in 3T3-L1 adipocytes and diabetic mice [44]. This finding implies that QG improves glucose absorbance in myoblasts by activating AMPK and insulin dependent Akt signaling via PTP inhibition.

QG promoted Akt phosphorylation in the association of insulin, even when palmitic acid was administered to mature myoblasts, indicating its potential to mitigate palmitic acid-induced insulin resistance (Figure 6). Insulin resistance, primarily attributed to T2DM, is closely linked with metabolic disorders, nonalcoholic fatty liver disease (NFALD), and obesity [45,46]. Aberrant lipid synthesis and compromised disposal processes may contribute to insulin resistance in muscle cells. It has been previously reported that increased free fatty acid levels result in the saturation of intracellular lipids predominantly diacylglycerol (DAG) and ceramide, disrupting insulin-Akt signaling and reducing glucose utilization [47]. A few previous studies have demonstrated that natural compound extracts, such as those from *Caulerpa lentillifera* and Black Quinoa, alleviate insulin resistance through the PI3K/Akt pathway in diabetic mice and HepG2 cells, respectively [48,49]. Hence, supporting our hypothesis, QG may serve as a drug to alleviate insulin resistance through the activation of insulin-Akt signaling.

Our study has a few limitations. Although we confirmed the anti-diabetic effect of QG in C2C12 muscles, a common in vitro model for such studies [50,51], it would be valuable to assess the influence of QG on lipid buildup or differentiation in 3T3-L1 adipocytes, considering the association of T2DM treatments with obesity [36,52]. In addition, various intermediate molecular markers, such as AS160, GLUT-4, and DAG, could be used to further support our study. Comprehensive in vivo studies are necessary to understand the systemic impact of QG in T2DM.

## 5. Conclusions

QG was recognized as a dual-target drug compound with an inhibitory effect against PTPN6 and PTPN9, presenting a potential treatment for diabetes in muscle cells. Treatment with QG stimulated glucose uptake by inhibiting PTPN6 and PTPN9, which further activated AMPK and insulin dependent Akt signaling in C2C12 cells. Furthermore, it alleviated insulin resistance induced by free fatty acids through the stimulation of Akt signaling. This study highlights QG as a potent and novel compound against diabetes that targets both PTPN6 and PTPN9.

## Figures and Tables

**Table 1:** Kinetic constants for PTPN6 and PTPN9.

	[E] (nM)	*K*_M_ (μM)	*V*_max_ (µM min^−1^)	*k*_cat_ (min^−1^)	*k*_cat_/*K*_M_ (µM^−1^ min^−1^)
PTPN6	6	187.5	10.78	1.8 × 10^3^	9.7
PTPN9	0.05	157.4	2.316	4.6 × 10^4^	292.2

## Data Availability

All study data are provided in the manuscript and Supplementary Materials. Detailed methods and additional data are available upon request from the corresponding authors.

## Supplementary Materials

The following supporting information can be downloaded at: https://www.mdpi.com/article/10.3390/nu16050647/s1, Figure S1: Heat map showing the effect of 20 natural drug compounds on 20 different PTPs. Figure S2: Michalis-Menten and Lineweaver-Burk Plots for PTPN6, and Figure S3: Michalis-Menten and Lineweaver-Burk Plots for PTPN9.
