# Supplementary material for: Exploring the Anti-Diabetic Potential of Quercetagitrin through Dual Inhibition of PTPN6 and PTPN9
Source: Nutrients. 2024 Feb 25;16(5):647. doi: 10.3390/nu16050647 (PMC10934500; doi:10.3390/nu16050647)
Supplement: Supplementary file 1 [file nutrients-16-00647-s001.zip › nutrients-2872242-supplementary.pdf]

Supplementary Information

Quercetagitrin as a dual-targeting inhibitor of PTPN6 and PTPN9 exhibits anti-diabetic effects

Geetanjali B. Gone [1†], Geonhui Go [1†], Gibeom Nam [1], Woojoo Jeong [1], Hyemin Kim [1], Soah Lee [1,2*] and Sang J. Chung [1,2*]

1 Department of Biopharmaceutical Convergence, Sungkyunkwan University, Suwon 16419, Republic of Korea

2 School of Pharmacy, Sungkyunkwan University, Suwon 16419, Republic of Korea

* Author to whom correspondence should be addressed.

† These authors contributed equally to this work

List of contents

Figure S1. Heat map showing the inhibitory data for PTPs……………………………………2

Figure S2. PTPN6 Michalis-Menten plots and Lineweaver-Burk plots ……………………...3

Figure S3. PTPN9 Michalis-Menten plots and Lineweaver-Burk plots ……………………...4

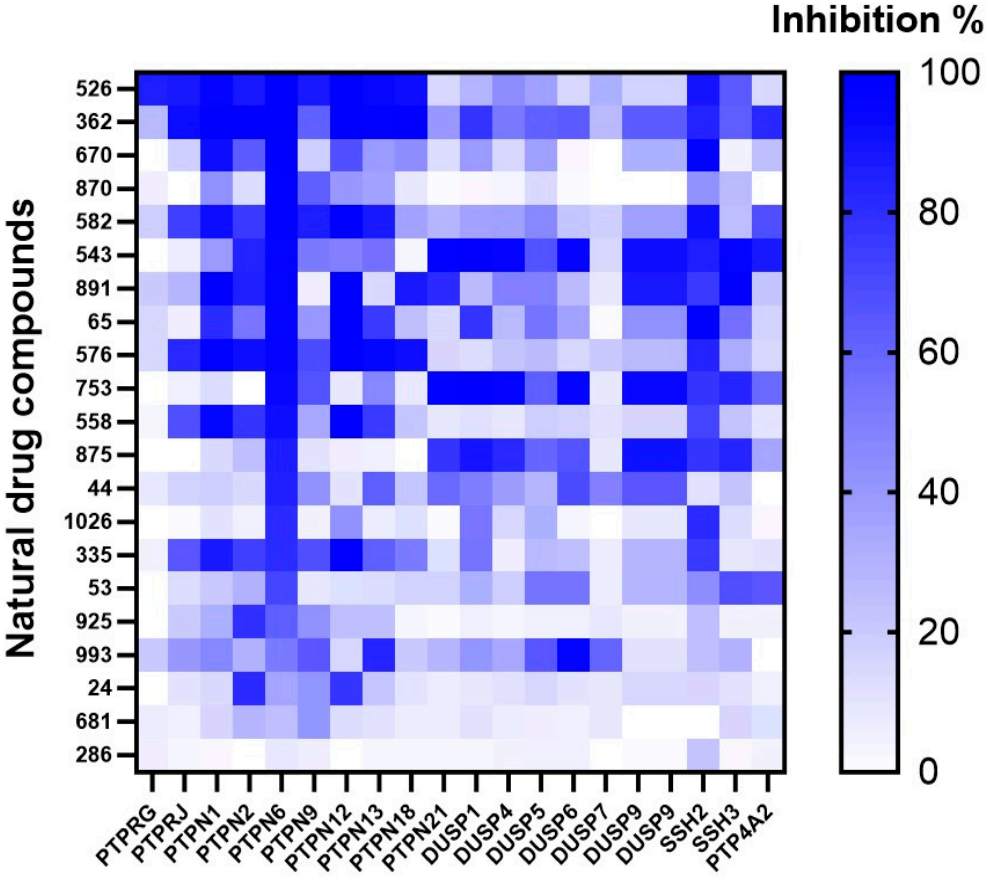

Figure S1: Heat map showing the effect of 20 natural compounds on 20 different PTPs. PTPs were added to solutions containing 20 μM of drug compounds in a reaction buffer with DiFMUP (2 × K_M) and catalytic activity was measured. Compound Number 870 represents Quercitagitrin.

(a)

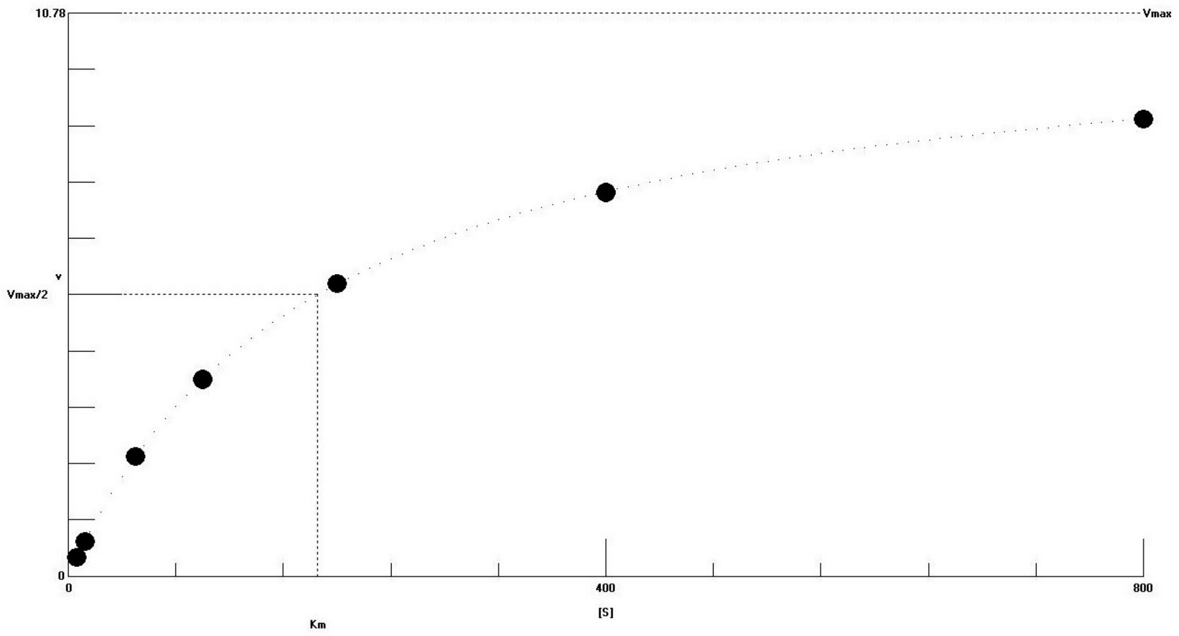

(b)

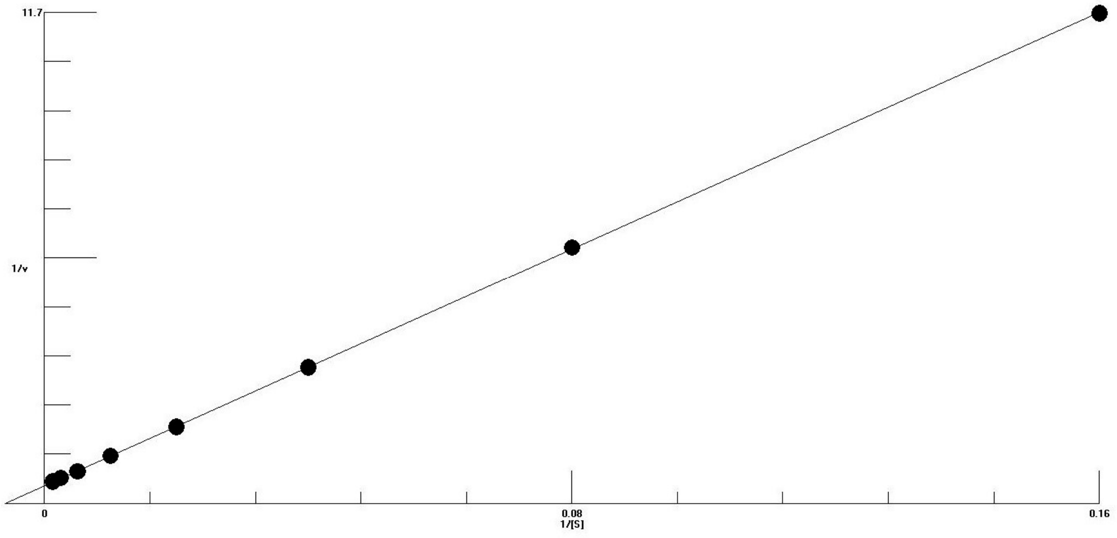

Figure S2. PTPN6 Michaelis–Menten plots (a) and Lineweaver–Burk plots (b) for the calculation of K_M and V_{max}. PTPN6 (6.0 nM) was added to the reaction buffer at various concentrations (800, 400, 200, 100, 50, 12.5, or 6.25 μM)

(a)

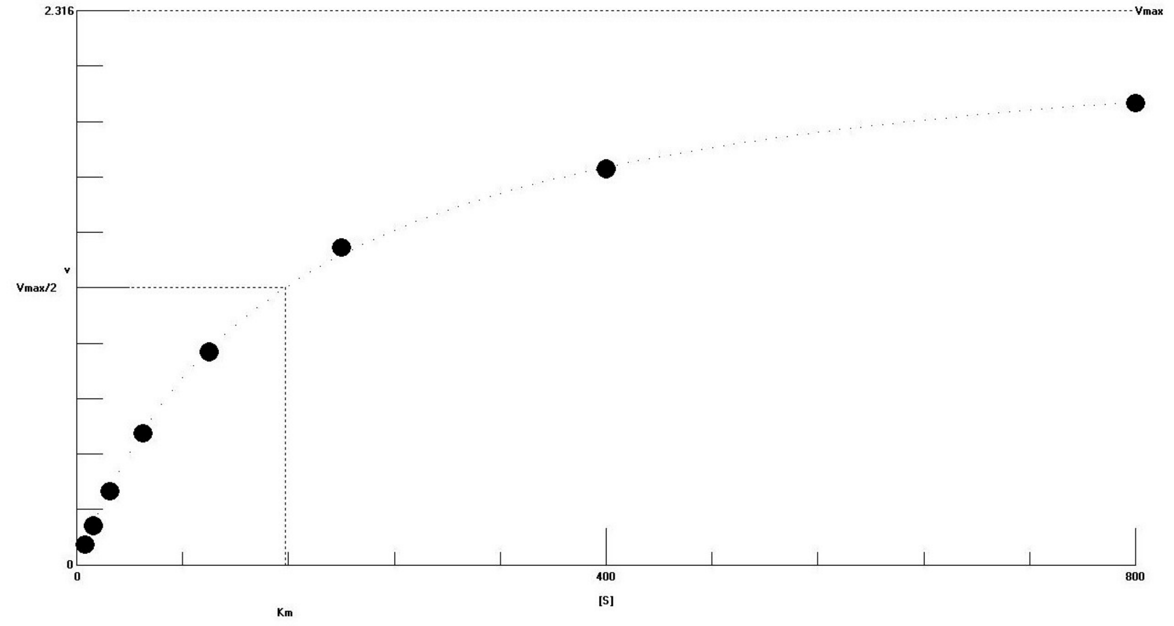

(b)

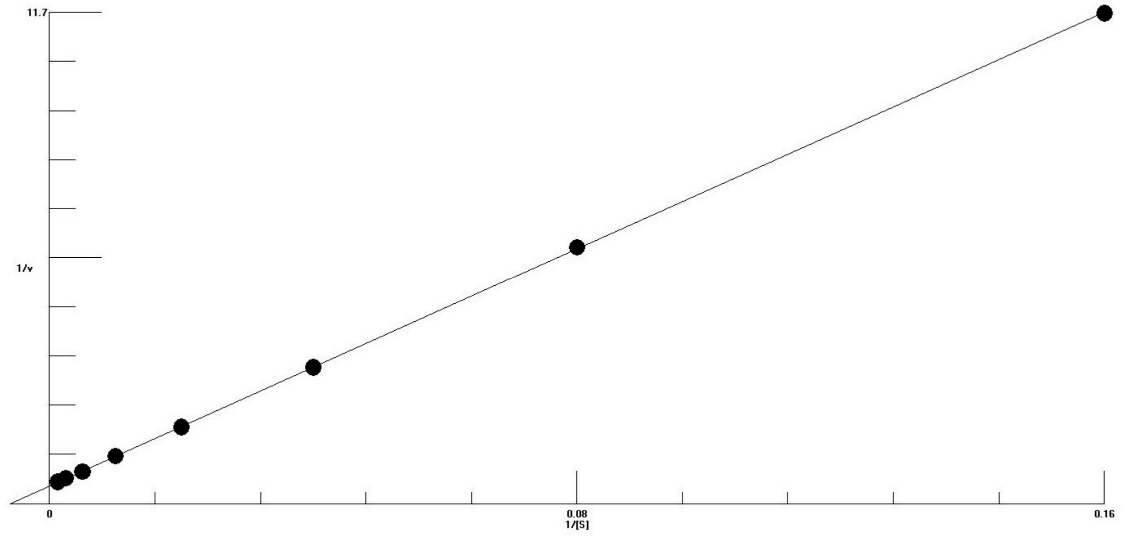

Figure S3. PTPN9 Michaelis–Menten plots (a) and Lineweaver–Burk plots (b) for the calculation of K_M and V_{max}. PTPN9 (0.05 nM) was added to the reaction buffer at various concentrations (800, 400, 200, 100, 50, 25, 12.5, or 6.25 μM)
